# MRI O-RADS: redefining diagnostic accuracy in characterization of ovarian lesions with histopathology as the benchmark

**DOI:** 10.1097/MS9.0000000000004217

**Published:** 2025-11-18

**Authors:** Zainab Nazir Sangi, Nasreen Naz, Samita Asad, Aneeqa Qureshi, Mumtaz Fareed, Syed Muhammad Shahnawaz Hyder, Ammar-E Yasir

**Affiliations:** a Dow Institute of Radiology, Dow University of Health Sciences (DUHS), Karachi, Pakistan; b Department of Radiology, The Indus Hospital, Karachi, Pakistan; c Department of Radiology, Shed Hospital, Karachi, Pakistan; d Radiologist, Mars Healthcare Network (Teleradiology Company), Pakistan

**Keywords:** adnexal masses, diagnostic accuracy, gynecologic malignancies, histopathology, magnetic resonance imaging, structured reporting

## Abstract

**Background:**

Adnexal lesions are a common gynecological concern requiring accurate distinction between benign and malignant types for optimal clinical management. The Ovarian-Adnexal Reporting and Data System for magnetic resonance imaging (MRI) (O-RADS MRI) aim to standardize risk assessment and enhance diagnostic precision.

**Objective:**

To evaluate the diagnostic accuracy of MRI using O-RADS MRI criteria, with histopathology as the gold standard.

**Methods:**

This descriptive cross-sectional study was conducted from January to June 2025 at the Dow Institute of Radiology, Dow University of Health Sciences (DUHS), Karachi, Pakistan. Women aged 18–65 years undergoing contrast-enhanced MRI for ultrasound-detected ovarian lesions, with available histopathology, were included. Patients with prior surgery, chemotherapy, radiotherapy, pregnancy, lactation, other malignancies, or inconclusive histopathology were excluded. O-RADS MRI scores were assigned by an experienced radiologist. Diagnostic metrics including sensitivity, specificity, predictive values, likelihood ratios, overall accuracy, and area under the curve (AUC) were calculated.

**Results:**

Among 145 patients (62.76% premenopausal, 37.24% postmenopausal), O-RADS categories 3 (32.41%) and 4 (31.03%) were most frequent. Benign lesions predominated in premenopausal women (60.44%), while malignant lesions were more common in postmenopausal women (66.67%). Common benign lesions included hemorrhagic cysts (14.48%), endometriomas (9.66%), and serous cystadenomas (6.90%), while serous cystadenocarcinoma (14.48%) was the most frequent malignancy. Using O-RADS ≥4 as the threshold for malignancy, sensitivity was 93.10%, specificity 79.31%, positive predictive value 75.00%, negative predictive value 94.52%, overall accuracy 84.83%, and AUC 0.921. Redefining O-RADS categories 3–5 as positive markedly reduced diagnostic accuracy to 56.42% and sensitivity to 66.67%

**Conclusion:**

The O-RADS MRI scoring system demonstrated excellent diagnostic accuracy in distinguishing benign from malignant ovarian lesions, supporting its role in clinical decision-making and risk stratification.

## Introduction

Gynecological cancers represent a significant global health burden, with ovarian cancer being one of the most lethal. Often referred to as a “silent killer,” ovarian cancer typically presents with vague, nonspecific symptoms such as abdominal discomfort, bloating, urinary frequency, fatigue, and altered bowel habits resulting in delayed diagnosis and poor prognosis. It ranks as the seventh most common cancer among women worldwide and is associated with a high mortality rate^[1,2]^.

According to the American Cancer Society, in 2018, approximately 22 240 new cases and 14 070 deaths due to ovarian cancer were reported in the United States alone^[3]^. Similarly, data from China in 2015 estimated 98 900 new ovarian cancer cases, placing it among the top gynecologic malignancies in women^[4]^. In Pakistan, the Karachi Cancer Registry reported one of the highest incidences of ovarian cancer in Asia, second only to urban Delhi^[5]^, with a 5-year survival rate of merely 46%^[6]^. Nationally, the incidence is increasing at an alarming rate of 13.6%, with nearly 70% of cases diagnosed at advanced stages^[6,7]^.

Survival rates for gynecologic cancers vary depending on cancer type, stage at diagnosis, and access to timely care. Late-stage detection significantly reduces survival chances, emphasizing the need for early diagnosis. Unfortunately, early detection of ovarian cancer remains a challenge due to the absence of specific symptoms and reliable biomarkers. This necessitates the development of rapid, noninvasive diagnostic tools^[1]^.

Imaging plays a crucial role in the evaluation of adnexal masses. Although ultrasound (especially transvaginal) remains the first-line imaging modality, its limitations such as operator dependency and limited field of view can compromise diagnostic accuracy^[7]^. In contrast, contrast-enhanced magnetic resonance imaging (MRI) offers superior soft tissue resolution, wider field of view, and is free of ionizing radiation. It enables better lesion characterization based on anatomical origin, shape, composition, and enhancement patterns^[8]^. While MRI improves diagnostic confidence, histopathology remains the gold standard for confirming the nature of adnexal lesions^[9]^.

The Ovarian-Adnexal Reporting and Data System for MRI (O-RADS MRI) was developed to standardize reporting and risk stratification of adnexal masses, categorizing lesions from 1 to 5 based on malignancy risk^[10]^. Recent studies have shown promising diagnostic performance for O-RADS MRI, with reported sensitivity and specificity as high as 92% and 85%, respectively^[11]^, and up to 94.83% and 87.50% in other analyses^[12]^. This study aims to assess the diagnostic accuracy of MRI in characterizing ovarian lesions using O-RADS MRI criteria, with histopathological findings as the reference standard. In addition, it evaluates the distribution of O-RADS categories and histopathological diagnoses across menopausal status and explores how diagnostic performance metrics vary when O-RADS category 3 is considered benign versus malignant. These insights may improve clinical decision-making, refine imaging protocols, and support early, accurate, and personalized management of adnexal masses.

## Material and methods

### Study design and setting

A descriptive cross-sectional study was conducted at the Dow Institute of Radiology, Dow University of Health Sciences (DUHS), Karachi, Pakistan.

### Sample size calculation

The sample size was estimated at 140 using the OpenEpi calculator, based on a disease prevalence of 13.6%^[13]^, sensitivity of 94.83%, specificity of 87.50%^[12]^, a 90% confidence interval, and a precision of <0.10.

### Participant selection

Adult female patients aged 18–65 years, referred for contrast-enhanced MRI following ultrasound-detected ovarian lesions and with available histopathology reports, were enrolled through consecutive sampling. Exclusion criteria included patients with inconclusive or unavailable histopathology, prior surgery, chemotherapy, or radiotherapy, pregnancy, lactation, or known malignancies other than ovarian.

**HIGHLIGHTS**

- The study calculates diagnostic performance of O-RADS MRI in characterizing adnexal lesions using histopathology as the reference standard.
- O-RADS MRI established high sensitivity (93.1%), specificity (79.3%), and AUC (0.921) for differentiating benign from malignant ovarian masses.
- Maintaining O-RADS category 3 as benign optimizes negative predictive value and reduces unnecessary surgical interventions.
- Differences in O-RADS category distribution between pre- and postmenopausal women highlight the importance of patient-specific risk assessment.
- The findings support broader implementation of O-RADS MRI, even in low-resource settings, through standardized training and contextual adjustments.

### MRI protocol and interpretation

MRI was performed on 1.5-Tesla scanners according to departmental protocol, including multiplanar and multi-sequential imaging sequences: T1-weighted imaging (T1WI), T2-weighted imaging (T2WI), short tau inversion recovery, diffusion weighted imaging (DWI), and post-contrast T1WI with phase contrast. Intravenous contrast was administered at 0.1 mL/kg body weight. All MRI scans were interpreted by a consultant radiologist with over 5 years of experience in women’s imaging. Adnexal lesions were evaluated and categorized using the O-RADS MRI (Supplemental Digital Content Table 1, available at: http://links.lww.com/JS9/F778) to assess malignancy risk^[14]^.

### Imaging and histopathological evaluation

The evaluation of adnexal lesions was conducted using a comprehensive MRI protocol, with particular focus on clinically relevant parameters including patient age, tumor size, presence of local invasion, wall and septal thickness, and enhancing solid components. Additionally, DWI signal intensity, presence of ascites, and lymph node involvement were systematically assessed to determine malignancy risk. Each of these radiological features was interpreted in accordance with the O-RADS MRI criteria. The final diagnostic confirmation for each case was established through histopathological examination, serving as the reference standard.

### Data analysis

Data were analyzed using SPSS version 22.0. Categorical variables (e.g., O-RADS category, histopathological diagnosis) were presented as frequencies and percentages, while continuous variables (e.g., age, symptom duration, tumor size) were summarized using median and interquartile range (IQR). With histopathology as the gold standard, diagnostic performance metrics of MRI were calculated, including sensitivity, specificity, positive predictive value (PPV), negative predictive value (NPV), overall accuracy, and area under the curve (AUC), using 2 × 2 contingency tables. A p-value less than 0.05 was considered statistically significant.

### Results

The study included 145 participants with a median age of 38 years (IQR: 21.00). Of these, 62.76% (*n* = 91) were premenopausal, and 37.24% (*n* = 54) were postmenopausal. The most commonly reported symptom was abdominal pain (88.97%, *n* = 129), followed by abdominal distention (51.03%, *n* = 74), amenorrhea (22.40%, *n* = 28), and postmenopausal bleeding (53.70%, *n* = 29). Menstrual cycle irregularities were highly prevalent, affecting 98.80% (*n* = 82) of the relevant subgroup. The median tumor volume was 318.24 cm^3^ (IQR: 925.77), and the median diameter was 8.47 cm (IQR: 6.83).

On MRI, unilocular cysts were observed in 24.83% (*n* = 36) and multilocular cysts in 72.41% (*n* = 105) of cases. Endometriotic cysts were identified in 8.16% (*n* = 8). T2 signal intensity was predominantly high (53.10%, *n* = 77), while T1 signal was mostly low (45.52%, *n* = 66), followed by high (34.48%, *n* = 50) and iso-intense signals (18.62%, *n* = 27). DWI (2/1) sequences were bright in 53.42% (*n* = 39). Additional MRI features included hemorrhage (31.96%, *n* = 31), lipid content (2.07%, *n* = 3), and presence of solid tissue (53.10%, *n* = 77). Thick enhancing septations were seen in 79.31% (*n* = 115), peritoneal/omental nodularity in 18.62% (*n* = 27), ascites in 26.21% (*n* = 38), and local invasion in 13.29% (*n* = 19).

Among 145 lesions, 60% were benign and 40% malignant. Malignant cases involved older, more often postmenopausal women and showed significantly larger tumor sizes. Clinically, amenorrhea and abdominal distention were more common. MRI features distinguishing malignancies included multilocular cysts, iso-intense T1 signals, solid components, thick enhancing septa, ascites, peritoneal/omental nodularity, and local invasion. Endometriotic cysts and hemorrhage were more frequent in benign lesions. Table 1 presents the detailed distribution and comparison of background characteristics and MRI features among the study participants stratified by histopathological diagnosis.

**Table 1:** Background characteristics and MRI features by final histopathological diagnosis

Background characteristics	Groups	Benign *n* = 87	Malignant *n* = 58	Total *n* = 145	*P-*value
Age [Median(IQR)]		34.00(16)	46.00(20.5)	38(21)	<0.001
Menopausal status, *n* (%)	Premenopausal	68 (78.16)	23 (39.66)	91 (62.76)	<0.001
	Postmenopausal	19 (21.84)	35 (60.34)	54 (37.24)	
Abdominal pain, *n* (%)	No	11 (12.64)	5 (8.62)	16 (11.03)	0.449
	Yes	76 (87.36)	53 (91.38)	129 (88.97)	
Amenorrhea, *n* (%)	No	71 (86.59)	26 (60.47)	97 (77.60)	<0.001
	Yes	11 (13.41)	17 (39.53)	28 (22.40)	
Cycle irregularity, *n* (%)	No	1 (1.92)	0 (0.00)	1 (1.20)	0.437
	Yes	51 (98.08)	31 (100.00)	82 (98.80)	
Abdominal distention, *n* (%)	No	60 (68.97)	11 (18.97)	71 (48.97)	<0.001
	Yes	27 (31.03)	47 (81.03)	74 (51.03)	
Postmenopausal bleeding, *n* (%)	No	10 (52.60)	15 (42.90)	25 (46.30)	0.492
	Yes	9 (47.40)	20 (57.10)	29 (53.70)	
Tumor size (volume) [Median(IQR)]		289.34(560.64)	366.69(1363.23)	318.24(925.77)	0.214
Tumor size (diameter)[Median(IQR)]		6.49(4.24)	11.83(5.83)	8.47(6.83)	<0.001
**MRI Feature**					
Unilocular Cyst, *n* (%)	No	58 (66.67)	51 (87.93)	109 (75.17)	0.004
	Yes	29 (33.33)	7 (12.07)	36 (24.83)	
Multilocular Cyst, *n* (%)	No	30 (34.48)	10 (17.24)	40 (27.59)	0.023
	Yes	57 (65.52)	48 (82.76)	105 (72.41)	
Endometriotic Cyst, *n* (%)	No	37 (84.09)	53 (98.15)	90 (91.84)	0.011
	Yes	7 (15.91)	1 (1.85)	8 (8.16)	
T2 signal, *n* (%)	Low	30 (34.48)	25 (43.10)	55 (37.93)	0.305
	High	51 (58.62)	26 (44.83)	77 (53.10)	
	Intermediate	2 (2.30)	2 (3.45)	4 (2.76)	
	Hetero	3 (3.45)	1 (1.72)	4 (2.76)	
	Dark	1 (1.15)	3 (5.17)	4 (2.76)	
	Bright	0 (0.00)	1 (1.72)	1 (0.69)	
T1 signal, *n* (%)	Low	42 (48.28)	24 (41.38)	66 (45.52)	<0.001
	High	42 (48.28)	8 (13.79)	50 (34.48)	
	ISO	1 (1.15)	26 (44.83)	27 (18.62)	
	Dark	2 (2.30)	0 (0.00)	2 (1.38)	
DWI 2/1 Signal, *n* (%)	Dark	22 (31.88)	2 (50.00)	24 (32.88)	0.809
	Bright	37 (53.62)	2 (50.00)	39 (53.42)	
	Intermediate	9 (13.04)	0 (0.00)	9 (12.33)	
	High	1 (1.45)	0 (0.00)	1 (1.37)	
Hemorrhage, *n* (%)	No	24 (54.55)	42 (79.25)	66 (68.04)	0.009
	Yes	20 (45.45)	11 (20.75)	31 (31.96)	
Lipid content, *n* (%)	No	86 (98.85)	56 (96.55)	142 (97.93)	0.341
	Yes	1 (1.15)	2 (3.45)	3 (2.07)	
Solid tissue, *n* (%)	No	64 (73.56)	4 (6.90)	68 (46.90)	<0.001
	Yes	23 (26.44)	54 (93.10)	77 (53.10)	
Thick enhancing septa, *n* (%)	No	26 (29.89)	4 (6.90)	30 (20.69)	<0.001
	Yes	61 (70.11)	54 (93.10)	115 (79.31)	
Peritoneal, mesenteric, omental nodularity, *n* (%)	No	87 (100.00)	31 (53.45)	118 (81.38)	<0.001
	Yes	0 (0.00)	27 (46.55)	27 (18.62)	
Ascites, *n* (%)	No	79 (90.80)	28 (48.28)	107 (73.79)	<0.001
	Yes	8 (9.20)	30 (51.72)	38 (26.21)	
Local invasion, *n* (%)	No	87 (100.00)	37 (66.07)	124 (86.71)	<0.001
	Yes	0 (0.00)	19 (33.93)	19 (13.29)	

Benign lesions comprised the majority, with hemorrhagic cysts (14.48%), endometriomas (9.66%), and serous cyst adenomas (6.90%) being most common. Among malignant lesions, serous cystadenocarcinoma (14.48%), germ cell tumors (5.52%), and mucinous cystadenocarcinoma (4.83%) were notable. Benign lesions clustered in O-RADS 2.00–3.00, especially hemorrhagic cysts and endometriomas, while malignant lesions were concentrated in O-RADS 4.00–5.00, notably serous cystadenocarcinomas and germ cell tumors. Full distributions by diagnosis and O-RADS category are detailed in Table 2 and Supplemental Digital Content Table 2, available at: http://links.lww.com/JS9/F778.

**Table 2:** Final diagnosis on histopathology of the study participant

Diagnosis	*n* (%)
Benign	
Abscess	2 (1.38)
Benign seromucinous cystadenoma	2 (1.38)
Complex hemorrhagic cyst	1 (0.69)
Complex ovarian cyst	4 (2.76)
Dermoid cyst	4 (2.76)
Endometrioma	14 (9.66)
Follicular retention cyst	7 (4.83)
Hemorrhagic cyst	21 (14.48)
Mucinous cyst adenoma	5 (3.45)
Paraovarian cyst	3 (2.07)
Ruptured ectopic with hematoma	2 (1.38)
Serous cyst adenoma	10 (6.90)
Simple ovarian cyst	5 (3.45)
Torsion	5 (3.45)
Tubo-ovarian complex infection	2 (1.38)
**Malignant**	
Brenner tumor	3 (2.07)
Clear cell carcinoma	2 (1.38)
Dermoid cyst with malignant transformation	2 (1.38)
Dysgerminoma	3 (2.07)
Endometroid carcinoma	6 (4.14)
Germ cell tumor	8 (5.52)
Krukenburg	1 (0.69)
Metastatic carcinoma	1 (0.69)
Mucinous borderline malignant	2 (1.38)
Mucinous cyst adenocarcinoma	7 (4.83)
Serous cystadenocarcinoma	21 (14.48)
Stromal tumor	1 (0.69)
Teratocarcinoma with peritoneal involvement	1 (0.69)

In this study, 17.0% of lesions (*n* = 26) were classified as O-RADS 2, all benign, indicating a 0.00% malignancy rate. O-RADS 3 accounted for 32.4% (*n* = 47), mostly benign, but included four malignant cases (8.51% malignancy rate). O-RADS 4 comprised 31.0% (*n* = 45) with a 60.00% malignancy rate, while O-RADS 5 (18.6%, *n* = 27) included only malignant lesions, showing a 100.00% malignancy rate. The distribution of benign and malignant diagnoses across O-RADS categories was statistically significant (*P* < 0.001). Detailed frequencies, histopathological outcomes, and malignancy rates are summarized in Table 3.

**Table 3:** Distribution of O-RADS MRI scores by final histopathological diagnosis

O-RADS MRI score	Benign, *n* (%)	Malignant, *n* (%)	Total, *n* (%)	Malignancy rate %	*P*-value
2.00	26 (29.89)	0 (0.00)	26(17.0)	0.00	<0.001
3.00	43 (49.43)	4 (6.90)	47(32.4)	8.51	
4.00	18 (20.69)	27 (46.55)	45(31.0)	60.00	
5.00	0 (0.00)	27 (46.55)	27(18.60)	100.00	

Among the total cases, 73 (50.30%) were classified as O-RADS 1–3, including 69 benign (79.31%) and 4 malignant (6.90%) lesions. The remaining 72 cases (49.70%) fell under O-RADS 4–5, comprising 54 malignant (93.10%) and 18 benign (20.69%) diagnoses. Diagnostic performance showed a sensitivity of 93.10% and specificity of 79.31%. The positive and negative likelihood ratios were 4.50 and 0.09, respectively. Disease prevalence was 40.00%, with a PPV of 75.00% and a NPV of 94.52%. Overall accuracy was 84.83%. Full details are provided in Table 4. Detailed distributions of MRI morphologic characteristics across O-RADS MRI categories are provided in Supplemental Digital Content Table 3, available at: http://links.lww.com/JS9/F778, illustrating the relationship between imaging features and malignancy risk.

**Table 4:** Diagnostic performance of O-RADS MRI score in differentiating benign and malignant lesions

O-RADS MRI score	Final diagnosis		Total, *n* (%)
	Benign, *n* (%)	Malignant, *n* (%)	
O-RADS benign (1–3)	69 (79.31)	4 (6.90)	73 (50.30)
O-RADS malignant (4–5)	18 (20.69)	54 (93.10)	72 (49.70)
Diagnostic matric	**Value**	**(95% CI)**	
Sensitivity	93.10	(83.27–98.09)	
Specificity	79.31	(69.29–87.25)	
Positive likelihood ratio	4.50	(2.96–6.83)	
Negative likelihood ratio	0.09	(0.03–0.23)	
Disease prevalence	40.00	(31.96–48.46)	
Positive predictive value	75.00	(66.40–81.99)	
Negative predictive value	94.52	(86.95–97.81)	
Accuracy	84.83	(77.93–90.24)	
*P*-value	<0.001		

**Figure 1.**
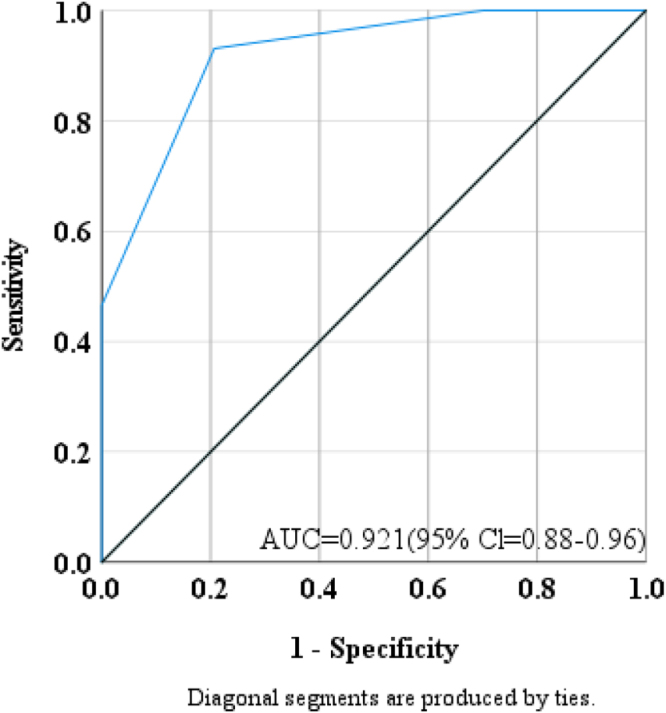
Receiver operating characteristic (ROC) curve for O-RADS MRI score in predicting malignancy.

The ROC curve analysis (Fig. 1) demonstrated excellent diagnostic performance of the O-RADS MRI score in differentiating malignant from benign adnexal lesions. The AUC was 0.921 (95% CI: 0.88–0.96; *P* = 0.000), confirming high discriminatory ability. The analysis included 58 malignant and 87 benign cases. Minor bias from tied results was noted. The optimal cutoff (>3.5) yielded a sensitivity of 93.1% and specificity of 79.3%, reflecting a strong diagnostic balance.

**Figure 2.**
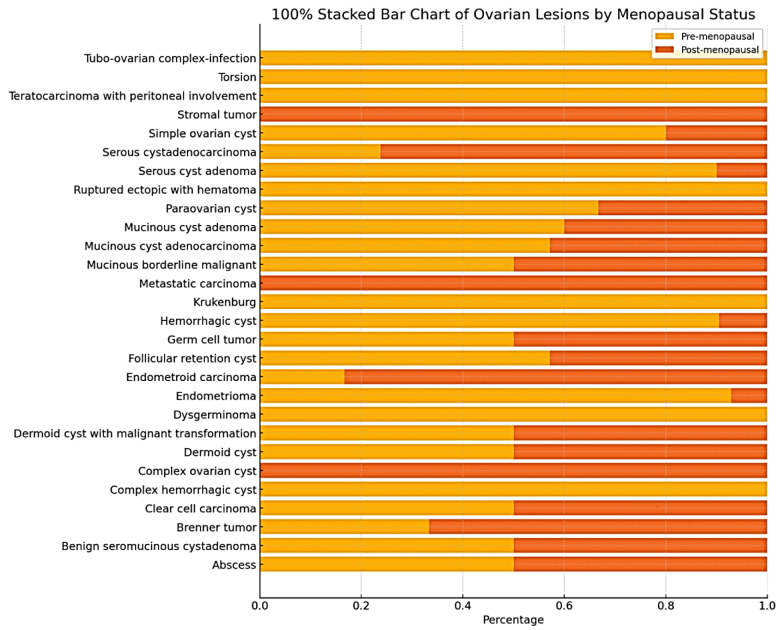
Final histopathological diagnoses by menopausal status.

Figure 2 presents O-RADS MRI categories and histopathological findings by menopausal status. O-RADS 3 (32.41%) and 4 (31.03%) were most common overall. Premenopausal women showed more benign classifications (60.44%) than postmenopausal (33.33%), while malignant scores were more frequent in postmenopausal (66.67%) than premenopausal (39.56%). Hemorrhagic cysts (14.48%) were the most frequent benign lesions, more common in premenopausal women (20.88%). Serous cystadenocarcinoma was the most common malignancy (14.48%), particularly among postmenopausal women (29.63%). Other notable malignancies in postmenopausal women included endometroid carcinoma (9.26%) and mucinous cystadenocarcinoma (5.56%).

Classifying O-RADS 3 as benign (positive test: O-RADS 4–5) yielded sensitivity of 93.10%, specificity of 79.31%, PPV 75.00%, NPV 94.52%, and accuracy 84.83%. Reclassifying O-RADS 3 as malignant (positive test: O-RADS 3–5) reduced sensitivity to 66.67%, specificity to 51.41%, PPV to 40.34%, NPV to 75.76%, and accuracy to 56.42%. Youden’s Index was higher when O-RADS 3 was treated as benign (0.72 vs. 0.18), confirming O-RADS 4 as the optimal malignancy threshold as shown in Table 5.

**Table 5:** Comparison of diagnostic performance metrics for O-RADS MRI scoring when category 3 is classified as benign versus malignant

Diagnostic metric	Scenario A (3 = Benign)	Scenario B (3 = Malignant)
Sensitivity	93.10%	66.67%
Specificity	79.31%	51.37%
Positive likelihood ratio	4.50	1.37
Negative likelihood ratio	0.09	0.65
PPV	75.00%	40.34%
NPV	94.52%	75.76%
Accuracy	84.83%	56.42%
Youden’s Index	0.72	0.18

Representative MRI images illustrating O-RADS 2–5 categories and corresponding histopathological diagnoses are presented in Supplemental Digital Content Figure 1, available at: http://links.lww.com/JS9/F778

## Discussion

This study demonstrates that using O-RADS categories 4 and 5 as the threshold for malignancy yields a sensitivity of 93.10%, specificity of 79.31%, and diagnostic accuracy of 84.83%. Notably, all lesions assigned to O-RADS 5 were confirmed malignant, underscoring its strong predictive value for high-risk cases. These findings align with Hassan *et al*, who reported a sensitivity of 92.3%, specificity of 82.6%, and accuracy of 86.1%, with 36.1% of masses classified as O-RADS 5^[15]^. The system’s high sensitivity reinforces its utility in guiding timely surgical intervention for suspected malignancies.

Consistent trends are observed when comparing with larger studies. Rizzo *et al* reported pooled sensitivity and specificity of 92% and 91%, respectively^[16]^, while Thomassin-Naggara *et al* also found 93% sensitivity and 91% specificity^[17]^. Kiliccap reported similar pooled values (sensitivity 93.0%, specificity 90.4%)^[18]^. Although our study’s specificity (79.31%) is comparatively lower suggesting more false positives its sensitivity (93.10%) remains aligned with prior findings. The higher malignancy prevalence in our cohort (40.0% vs. 21.6% in Rizzo *et al*)^[16]^ may explain this variation. Basu *et al*’s cancer prediction model, with higher specificity (87.8%) and AUC of 0.962, suggests added tools may reduce false positives^[19]^.

The reliability of O-RADS scores 4 and 5 in our study is further supported by findings from Takkar *et al*, who reported high specificity for malignancy at 89% and 88%, respectively. Categories 2 and 3 showed perfect sensitivity but low specificity for benign lesions, reinforcing the system’s value in accurately excluding malignancy and prioritizing high-risk lesions for further clinical action^[20]^.

This study identified malignancy-associated features such as ascites, solid components, enhancement, peritoneal nodularity, and multilocular cysts aligned with findings by Wengert *et al*^[21]^ and Basu *et al*^[19]^. Malignancies were more prevalent in postmenopausal women, especially serous cystadenocarcinoma and stromal tumors, while benign lesions like hemorrhagic and endometriotic cysts predominated in premenopausal women. Distinct malignancies, including Krukenberg tumors, were noted among premenopausal patients. These results highlight how crucial it is to combine imaging with clinical presentation and patient demographics in order to make an accurate diagnosis.

Reclassifying O-RADS 3 lesions as malignant significantly impaired diagnostic performance, resulting in a marked decrease in specificity (from 79.31% to 51.41%), overall accuracy (from 84.83% to 56.42%), and sensitivity (from 93.10% to 66.67%). PPV fell to nearly 40%, increasing false positives, while NPV also declined. Findings support keeping O-RADS 3 as benign, consistent with international guidelines. Thomassin-Naggara *et al* and Sadowski *et al*, showed that classifying O-RADS 3 as benign preserves NPV and reduces overtreatment^[14,17]^. Our findings align with these, supporting that maintaining O-RADS 3 as “negative” enhances diagnostic accuracy and adheres to international guidelines.

Our study demonstrated high diagnostic accuracy of the O-RADS MRI scoring system in differentiating benign from malignant adnexal lesions, with a sensitivity of 93.1%, specificity of 79.3%, and an AUC of 0.921 using a threshold of ≥4. These findings are consistent with previous research reporting similarly high performance metrics, reinforcing the clinical utility of this threshold^[22]^. Maintaining O-RADS 3 as a benign category supports optimal accuracy and avoids unnecessary interventions. However, further validation is needed. A well-designed prospective, multicenter study with a larger sample size and specific focus on indeterminate adnexal masses is recommended to refine diagnostic accuracy and clinical applicability.

One of O-RADS MRI’s main advantages is its reproducibility across different radiological skill levels. Cabedo *et al* demonstrated that with standardized training, even less experienced radiologists achieved high diagnostic accuracy. This suggests that O-RADS MRI can be effectively implemented in resource-limited settings like Pakistan^[23]^, promoting consistent diagnostic standards and enhancing patient care across diverse clinical environments.

O-RADS MRI shows high sensitivity (93.10%) in detecting malignant ovarian lesions, making it valuable in settings like Pakistan, where ovarian cancer is prevalent. Its utility is evident as all O-RADS 5 cases were confirmed malignant. However, its lower specificity (79.31%), compared to studies like Rizzo *et al*^[16]^ and Thomassin-Naggara *et al*^[17]^, suggests a higher false-positive rate, potentially leading to unnecessary surgeries. This emphasizes the need for context-specific refinements, such as integrating time-intensity curve analysis^[21]^ or predictive models^[19]^. Additionally, differences by menopausal status highlight the importance of tailored diagnostic approaches, especially in low-resource settings with delayed presentations due to socioeconomic constraints.

This study benefits from using histopathology as the gold standard and a diverse patient population from a tertiary care center. However, its limitations include a single-center design, small sample size (145 patients), and selection bias due to inclusion of only surgically managed cases. Unlike larger multicenter studies (e.g., Rizzo *et al*, Thomassin-Naggara *et al*), this may reduce generalizability. Interobserver variability, not assessed here, remains a concern. Future studies should be multi-institutional, especially in low-resource settings, incorporate standardized O-RADS training, evaluate interobserver agreement, and integrate advanced imaging (e.g., Time-Intensitiy Curve (TIC) analysis) or prediction models to improve specificity and reduce unnecessary surgeries.

## Conclusion

O-RADS MRI offers high diagnostic accuracy for adnexal mass evaluation using histopathology as the gold standard. A cutoff of ≥4 yields excellent sensitivity and acceptable specificity, supporting its clinical utility. Retaining O-RADS 3 as a benign category helps avoid unnecessary surgeries. Differences in score distribution between pre- and postmenopausal women highlight the importance of considering menopausal status in risk stratification. Overall, O-RADS MRI is a reliable, standardized tool for distinguishing benign from malignant lesions, enabling more accurate decision-making, optimizing patient management, and improving outcomes through timely referral for high-risk cases and conservative management for low-risk ones.

## Data Availability

The data supporting this study are available from the corresponding author upon reasonable request. The data are not publicly available due to privacy or ethical considerations.

## Research registration unique identifying number (UIN)

RTMC Registration No: RAD-2022-256-3756.

## Peer and provenance statement

Not commissioned, externally peer-reviewed.
